# Metabolites as regulators of autoimmune diseases

**DOI:** 10.3389/fimmu.2025.1637436

**Published:** 2025-08-21

**Authors:** Maria Tada, Michihito Kono

**Affiliations:** Department of Rheumatology, Endocrinology and Nephrology, Faculty of Medicine and Graduate School of Medicine, Hokkaido University, Sapporo, Japan

**Keywords:** cellular metabolism, metabolite, itaconate, glycolysis, glutaminolysis

## Abstract

Immune cell metabolism is essential for regulating immune responses, including activation, differentiation, and function. Through glycolysis and oxidative phosphorylation (OXPHOS), metabolism supplies energy and key intermediates for cell growth and proliferation. Importantly, some metabolites generated during these processes act as signaling molecules that influence immune activity. Autoimmune diseases such as rheumatoid arthritis (RA) and systemic lupus erythematosus (SLE) involve multiple immune cell types, and recent research in immunometabolism has revealed that disrupted metabolic pathways in these cells contribute to disease progression. Effector T cells, for instance, undergo metabolic reprogramming, particularly increased glycolysis, to meet the demands of proliferation and function during autoimmune responses. Targeting metabolic enzymes has shown therapeutic potential. In addition, metabolites themselves, termed immunometabolites, can directly modulate immune responses. These include both intracellularly generated and secreted molecules. Itaconate is a key immunometabolite and is derived from the TCA cycle by aconitate decarboxylase 1 (ACOD1) in activated macrophages. It inhibits the NLRP3 inflammasome and pro-inflammatory cytokines, such as IL-1β and IL-6. Beyond macrophages, itaconate alters metabolism and epigenetics in T cells by reducing 2-hydroxyglutarate and the S-adenosyl-L-methionine (SAM)/S-adenosyl-L-homocysteine (SAH) ratio, thereby suppressing Th17 differentiation and enhancing Foxp3 expression in Tregs. Itaconate ameliorates disease in experimental autoimmune encephalomyelitis, RA, SLE, and others. It also exhibits antimicrobial effects by blocking bacterial isocitrate lyase and viral replication. Despite increasing interest, reviews focusing specifically on immunometabolites remain limited. This review highlights emerging insights into metabolites involved in glycolysis, the TCA cycle, glutaminolysis, one-carbon metabolism, and lipid metabolism that influence autoimmune pathophysiology.

## 1 Introduction

Metabolism plays a crucial role in regulating the function of immune cells, influencing their activation, differentiation, and overall immune responses (1). It is essential for producing adenosine triphosphate (ATP) via several pathways, such as glycolysis and oxidative phosphorylation (OXPHOS). Metabolism also provides essential intermediates for nucleotide, amino acid, and lipid synthesis during cell proliferation and activation. Importantly, metabolites produced through intracellular metabolic processes can act as signaling molecules that regulate various cellular activities (2, 3).

Recently, immunometabolism, the study of metabolism in immune cells, has revealed that dysregulated immune cell metabolism contributes to the pathophysiology of autoimmune diseases (4). Autoimmune diseases, such as rheumatoid arthritis (RA) and systemic lupus erythematosus (SLE), involve multiple different types of immune cells. Autoimmune diseases are characterized by a loss of tolerance to self-antigens, resulting in chronic inflammation and tissue damage. This breakdown of self-tolerance involves multiple immune cell subsets, including autoreactive effector T cells, such as Th1 and Th17, dysfunctional Tregs, activated B cells, and pro-inflammatory macrophages (5). These immune cells contribute to the excessive production of cytokines, autoantibodies, and tissue-infiltrating inflammatory mediators, thereby promoting disease progression (5). For instance, T cells rely on metabolic reprogramming, particularly glycolysis, for their energetic and biosynthetic demands during proliferation and differentiation into effector T cells (6). This metabolic shift is crucial for their activation and subsequent functions, especially in the context of autoimmune diseases (7). Therefore, the targeting of specific enzymes in immune cells is gaining traction as a potential therapeutic strategy (8). Moreover, recent research has also demonstrated that certain metabolites themselves influence immune cell responses by either accumulating within immune cells or by being secreted by non-immune cells to modulate immune responses (**Table 1**). These metabolites are defined as immunometabolites, and their modulation or supplementation could also be a new therapeutic approach.

**Table 1:** Immunomodulatory functions of key metabolites in autoimmune and inflammatory diseases.

Metabolite	Main pathway	Cell type	Function	Associated diseases
Lactate	Glycolysis	CD4+ T cells	Induces Th17 differentiation	RA, MS, Sjögren’s syndrome
		Macrophages	Promotes immunosuppressive genes	
		B cells	Induces proinflammatory senescence	
Pyruvate	Glycolysis	T cells	Anti-inflammatory effects, inhibits HMGB1, promotes Tregs	Myocarditis, Encephalomyelitis, T1D
		Macrophages	Reduces mitochondrial ROS and pro-inflammatory cytokines	
α-KG	TCA cycle	T cells	Promotes Treg, inhibits Th17	RA, SLE
		Macrophages	Anti-inflammatory macrophage polarization	
Succinate	TCA cycle	Macrophages	Stabilizes HIF-1α, promotes IL-1β/NLRP3 activation	Experimental autoimmune uveitis, Arthritis
		T cells	Th1/Th17 differentiation, protein succinylation	
		Dendritic cells	Influences DC maturation/migration	
Fumarate (DMF)	TCA cycle	Macrophages	Promotes M2 macrophages, reduces IL-1β and IL-6 by blocking NF-kB nuclear translocation and ERK1/2-MSK1 signaling	EAE, Psoriasis
		Dendritic cells	Impairs maturation by downregulating MHC class II, CD80/86, and inflammatory cytokine production (IL-12, IL-6)	
Malate	TCA cycle	Macrophages	Reduces IL-1β, TNFa, and IL-6 by modulating the BiP-IRF2BP2 signaling pathway	IBD, RA
Itaconate	TCA cycle	Macrophages	Inhibits SDH, reduces ROS/cytokines, modulates Nrf2/STING/NLRP3	SLE, RA, EAE, IBD
		T cells	Suppresses Th17, enhances Tregs by suppressing glycolysis and OXPHOS and modulating epigenetic reprogramming	
		FLS	Inhibits glycolysis/OXPHOS, inhibits proliferation and migration	
Acetate	TCA cycle	T cells	Supports T cell effector function under glucose restriction, contributes to acetyl-CoA pool	EAE, IBD
Glutamine	Amino acid metabolism	CD4 T cells	Promotes Th1/Th17 differentiation, suppresses Tregs	AIH, SLE, RA
Glutamate	Amino acid metabolism	T cells	Promotes Th17 differentiation	EAE, SLE,
Aspartate	Amino acid metabolism	T cells	Maintains ER homeostasis, suppresses TNF release	RA
		Macrophages	Promote HIF-1α stabilization and IL-1β	
Methionine	One-carbon metabolism	CD4 T cells	Promote Th17 differentiation	EAE
SAM/SAH	One-carbon metabolism	CD4 T cells	Maintains H3K4 methylation, promotes Th17 differentiation	MS
25-OHC	Lipid metabolism	CD4 T cells	Suppresses cholesterol biosynthesis, induces apoptosis in proliferating T cells	Contact hypersensitivity, EAE
S1P	Lipid metabolism	T cells	Regulates T cell egress (via S1PR1)	MS, SLE, RA
		FLS	Promotes inflammation in RA-FLS	

This table summarizes key metabolites involved in immunometabolism, categorized by their main metabolic pathways. The listed metabolites exert cell type-specific effects on immune function, including T cell differentiation, macrophage polarization, and cytokine production. The associated autoimmune diseases reflect the pathological relevance of each metabolite’s activity.

α-KG, alpha-ketoglutarate; DMF, Dimethyl fumarate; SAM, S-adenosyl-L-methionine; SAH, S-adenosylhomocysteine; 25-OHC, 25-Hydroxycholeesterol; S1P, Sphingosine-1-phosphate; TCA, the tricarboxylic acid; ROS, reactive oxygen species; DC, Dendritic cells; FLS, fibroblast-like synoviocytes, RA, rheumatoid arthritis; MS, Multiple sclerosis; T1D, Type 1 diabetes; EAE, Experimental autoimmune encephalomyelitis; SLE, systemic lupus erythematosus; IBD, inflammatory bowel disease; AIH, autoimmune hepatitis.

While many review papers focusing on enzymes in immunometabolism have been published, those focused on immunometabolites are limited. The present review discusses the latest research on representative immunometabolites in glycolysis, the tricarboxylic acid (TCA) cycle, glutaminolysis, and lipid metabolism, which regulate the pathophysiology of autoimmune diseases, including systemic lupus erythematosus (SLE) and rheumatoid arthritis (RA).

## 2 Glycolysis

### 2.1 Lactate

**Figure 1:**
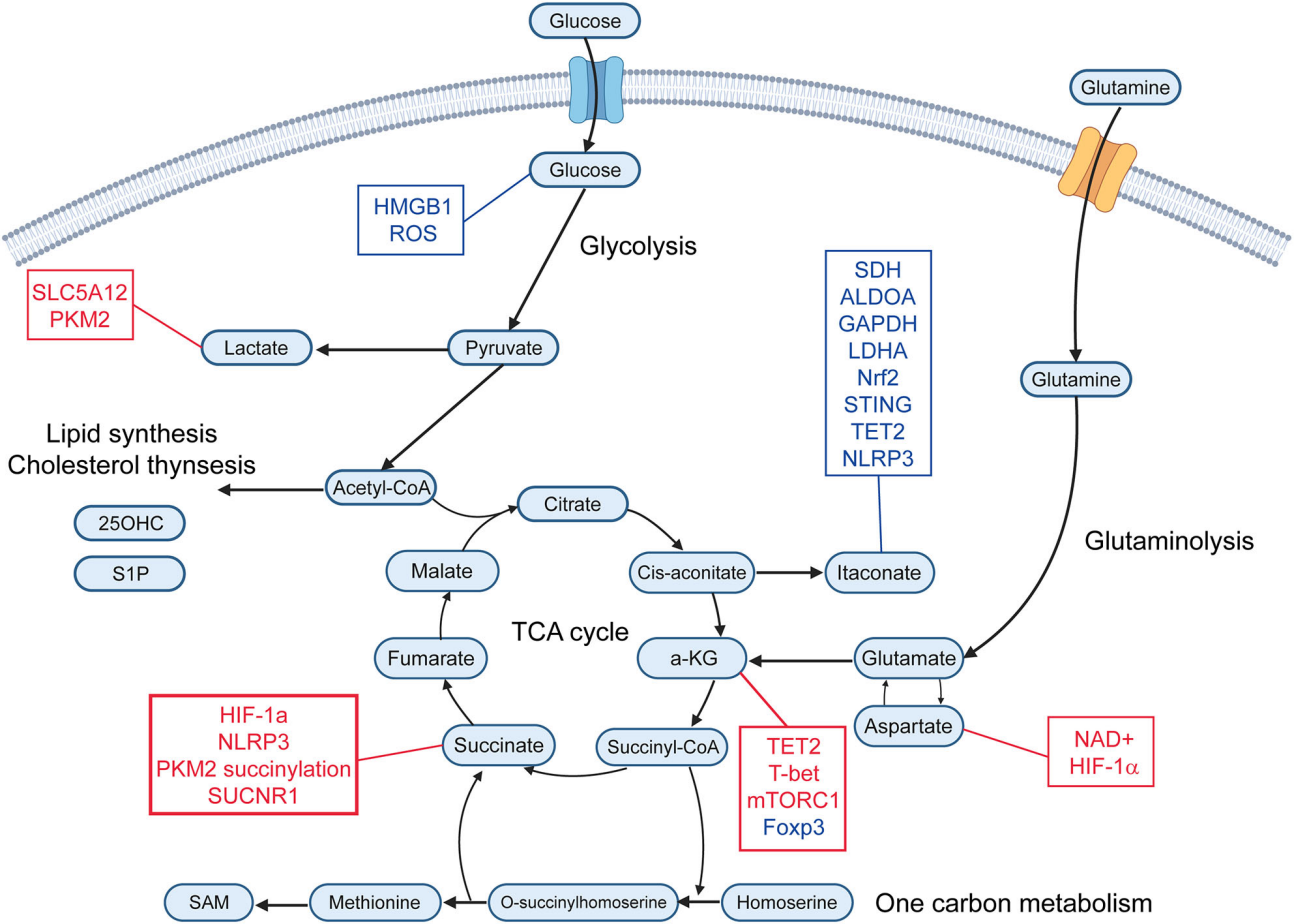
Immunometabolites and their molecular targets. This schematic illustrates key immunometabolites that regulate immune responses. Metabolites are organized according to their primary metabolic pathways. Immunomodulatory metabolites are connected to their downstream molecular targets. Red text represents upregulation or activation of the indicated target, while blue text represents downregulation or inhibition. α-KG, alpha-ketoglutarate; SAM, S-adenosyl-L-methionine; 25-OHC, 25-Hydroxycholeesterol; S1P, Sphingosine-1-phosphate; ROS, reactive oxygen species; PKM2, pyruvate kinase M2; SDH, succinate dehydrogenase; ALDOA, aldolase A; GAPDH, glyceraldehyde-3-phosphate dehydrogenase; LDHA, lactate dehydrogenase; Nrf2, nuclear factor erythroid 2-related factor 2; TET2, ten-eleven translocation 2.

Lactate is a metabolite generated from pyruvate by lactate dehydrogenase (LDH) through anaerobic glycolysis (**Figure 1**) (9). This pathway is activated under conditions of high energy demand or limited oxygen availability, such as during infection, inflammation, or cancer (10). In RA, lactate levels are markedly elevated in the hypoxic synovial microenvironment compared to osteoarthritis, primarily due to increased production and secretion by fibroblast-like synoviocytes (11). Elevated serum lactate levels have been reported in patients with multiple sclerosis and Sjögren’s syndrome (12, 13). Lactate exerts diverse immunomodulatory effects, functioning as both an immunosuppressive and pro-inflammatory mediator.

Lactate modulates T cell function, contributing to inflammatory processes. The accumulation of lactate leads to upregulation of the lactate transporter SLC5A12 in human CD4 T cells, resulting in lactate uptake into CD4 T cells (14). This induces nuclear translocation of dimeric pyruvate kinase M2 (PKM2), activates acetyl-CoA carboxylase, thereby suppressing glycolysis and promoting fatty acid synthesis (14, 15). Human CD4 T cells treated with lactate increased IL-17A production (14). Inhibition of PKM2 reduces Th17 cell differentiation and attenuates disease activity of experimental autoimmune encephalomyelitis (EAE) (9). Furthermore, blockade of SLC5A12 has been shown to alleviate disease severity in a murine model of arthritis (14). However, other studies have shown that lactate suppresses the Th17 cell differentiation and promotes Treg cell differentiation (16, 17). These discrepancies should be further investigated in future studies.

Recent studies have shown that aging B cells shift their cellular metabolism toward glycolysis, secrete increased amounts of lactate, and adopt a proinflammatory phenotype via the SLC5A12, characterized by senescence-associated profile mediators and the production of autoantibodies, such as anti-dsDNA (18).

**Figure 2:**
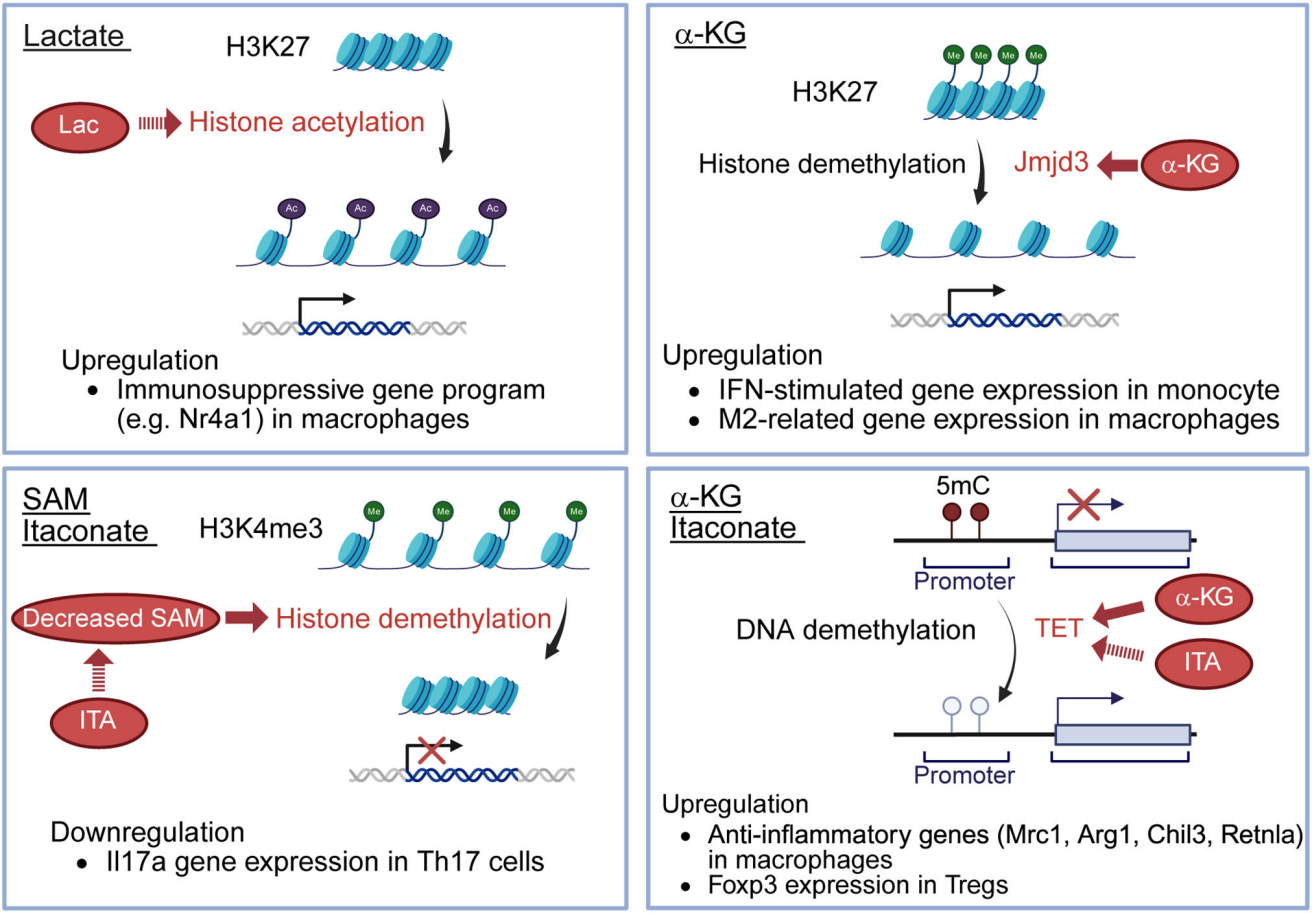
Effects of immunometabolites for epigenetics. Lactate serves as a fuel to promote histone H3K27 acetylation, resulting in the upregulation of immunosuppressive genes, such as Nr4a1, in macrophages. Itaconate decreases SAM/SAH ratio, thereby limiting histone demethylation at loci of the Il17a promoter, resulting in the downregulation of Il17a gene expression in Th17 cells. α-KG enhances the activity of Jmjd3, a histone demethylase, promoting histone H3K27 demethylation and inducing the upregulation of IFN-stimulated gene expression in monocytes and M2-related gene expression in macrophages. α-KG also serves as a cofactor for TET2, a DNA demethylase, leading to upregulated anti-inflammatory genes (Mrc1, Arg1, Chil3, Retnla) in macrophages. Itaconate indirectly promotes TET by inhibiting isocitrate dehydrogenases 1/2 in Tregs, decreasing 2-HG, known to inhibit TET enzymes. The increased TET activity allows TET-mediated DNA demethylation, leading to the upregulation of Foxp3 expression in Tregs. SAM, S-adenosyl-L-methionine; SAH, S-adenosyl-L-homocysteine; α-KG, alpha-ketoglutarate; TET2, ten-eleven translocation 2; 2-HG, 2-hydroxyglutarate; Lac, lactate; ITA, itaconate; 5mC, 5-methylcytosine.

On the other hand, in macrophages, recent research has revealed that lactate serves as a fuel to promote histone H3K27 acetylation, leading to epigenetic changes, enabling the expression of immunosuppressive genes, such as *Nr4A1* (19) (**Figure 2**). This process transcriptionally represses pro-inflammatory functions in macrophages and sustains long-term immunosuppression (19). These differences may reflect cell type-specific responses or variations in disease models.

### 2.2 Pyruvate

Pyruvate is a central metabolite produced primarily through glycolysis (**Figure 1**), wherein glucose is converted to pyruvate by pyruvate kinase under conditions of high energy demand, such as immune cell activation. Additional sources include lactate via LDH and alanine via alanine aminotransferase. Mitochondrial pyruvate uptake via the mitochondrial pyruvate carrier links glycolysis to the TCA cycle, supporting ATP production and biosynthetic pathways (20, 21). Ethyl pyruvate (EP), a stable derivative of pyruvate, has shown efficacy in experimental models of autoimmune diseases such as myocarditis, encephalomyelitis, and type 1 diabetes (22–24). EP reduces immune cell infiltration and inflammation by inhibiting HMGB1 signaling and promoting regulatory immune cells, including Tregs and tolerogenic dendritic cells (22, 24). In macrophages, pyruvate has anti-inflammatory effects by reducing mitochondrial reactive oxygen species (ROS) and pro-inflammatory cytokines, such as IL-1β, IL-6, and TNFα during viral infections (25).

## 3 TCA cycle

### 3.1 Succinate

Succinate, a key intermediate in the TCA cycle (**Figure 1**), is accumulated in human cells under conditions of hypoxia, inflammatory activation, and metabolic stress (26). In hypoxic microenvironments, macrophages shift toward glycolysis, leading to succinate accumulation due to impaired mitochondrial oxidation and reverse electron transport (27). Lipopolysaccharide (LPS) activation of Toll-like receptor 4 (TLR4) in macrophages drives succinate accumulation by inhibiting succinate dehydrogenase (SDH) and upregulating glutamine-dependent anaplerosis (27, 28). Inflammatory stimuli promote glycolytic flux, thereby increasing succinate production through the TCA cycle. Hypoxia-inducible factor-1α (HIF-1α) stabilizes under low oxygen, further enhancing glycolysis and succinate generation. Mitochondrial dysfunction (ROS overproduction) or mutations in SDH subunits can lead to pathological succinate accumulation (29).

Succinate accumulation in activated macrophages stabilizes HIF-1α by inhibiting prolyl hydroxylases, leading to increased transcription of IL-1β and NLRP3 inflammasome activation (27). High alpha-ketoglutarate (α-KG)/succinate ratios favor anti-inflammatory macrophage polarization, while low ratios drive pro-inflammatory responses (30). Cytosolic succinate can modify proteins, such as PKM2 via succinylation, enabling its nuclear translocation and transcriptional activation of pro-inflammatory genes in macrophages (31). Extracellular succinate binds the G-protein-coupled receptor SUCNR1 on anti-inflammatory macrophages, triggering calcium signaling and ERK phosphorylation to promote tissue repair and prostaglandin E2 secretion (32). In T cells, succinate directly influences their differentiation into pro-inflammatory Th1 and Th17 cells, especially under the condition of impaired SDH function and intracellular succinate accumulates. Elevated succinate/α-KG ratios in T cells upregulate pro-inflammatory gene expression and cytokine production, including IFNγ and IL-17A (33). In a mouse model of experimental autoimmune uveitis, succinate administration exacerbate disease severity by promoting neutrophil extracellular trap formation and increasing Th1/Th17 cell populations (34). Succinate can also suppress T cell degranulation and cytokine secretion in CD4 and CD8 T cells, particularly affecting the production of IFNγ (35). In dendritic cells, succinate promotes their maturation and migration to lymph nodes via SUCNR1, enhancing antigen presentation and IL-1β secretion, which facilitates T cell activation (36). In experimental arthritis models, succinate exposure increases Th17 cell frequencies and disease severity through SUCNR1-expressing dendritic cells (37).

### 3.2 Itaconate

ITA is one of the most important endogenous immunometabolites derived from the mitochondrial TCA cycle primarily in activated macrophages. ITA is produced from cis-aconitate (**Figure 1**), the TCA cycle intermediate, by aconitate decarboxylase 1 (ACOD1) which is induced during inflammatory responses in macrophages (38). ITA is also known to be produced in macrophages in response to glucocorticoid stimulation, and interestingly, this pathway is essential for the immunosuppressive action of glucocorticoids (39). ITA inhibits SDH, leading to reduced mitochondrial ROS, and suppression of pro-inflammatory cytokines, including IL-1β and IL-6 in macrophages (40). ITA and its derivatives also directly inhibit key glycolytic enzymes such as aldolase A (ALDOA), glyceraldehyde-3-phosphate dehydrogenase (GAPDH) and lactate dehydrogenase A (LDHA), thereby reducing glycolysis and inflammatory responses (41). Moreover, ITA modulates transcription factors, such as nuclear factor erythroid 2-related factor 2 (Nrf2), STING, ten-eleven translocation 2 (TET2) and NLRP3, reducing inflammasome activation and oxidative stress (42, 43). Recent research indicates that ITA affects not only macrophages but also other immune cells and non-immune cells. ITA suppresses glycolysis and oxidative phosphorylation in T cells, leading to metabolic and epigenetic reprogramming of T cells (44). ITA decreases Th17 cell differentiation and increases Treg cell differentiation (44). In contrast, ITA does not affect Th1 or Th2 cell differentiation (44). Following treatment with ITA, the S-adenosyl-L-methionine (SAM)/S-adenosylhomocysteine (SAH) ratio is decreased, reducing histone methylation at loci of the Il17a promoter and limiting RORγt binding at the Il17a promoter in Th17 cells (44) (**Figure 2**). ITA also inhibits isocitrate dehydrogenases 1/2 in Tregs, decreasing 2-hydroxyglutarate (44), known to inhibit TET enzymes (45). The upregulation of TET allows TET-mediated DNA demethylation, increasing Foxp3 expression in Tregs (44) (**Figure 2**). Moreover, ITA can ameliorate disease activity in an EAE model (44). ITA also reduces the proliferation and migration of fibroblast-like synoviocytes (FLS) and ameliorates arthritis by inhibiting glycolysis and mitochondrial OXPHOS (46). It has been reported that ITA and its derivatives also ameliorate mouse models of SLE (47), inflammatory bowel diseases (48), and lung fibrosis (49). ITA also exerts antimicrobial effects by direct inhibition of bacterial isocitrate lyase and suppressing viral replication (50). Therefore, ITA could be considered a novel therapeutic target for autoimmune diseases with a low risk of infection.

### 3.3 α-KG

α-KG is primarily produced in mitochondria via the TCA cycle (**Figure 1**), where isocitrate dehydrogenase catalyzes the conversion of isocitrate to α-KG, generating NADH. Additionally, α-KG is produced from glutamine through glutaminase and glutamate dehydrogenase or transaminases. This pathway, called anaplerosis, is especially active in proliferating cells. α-KG plays an important role in modulating macrophage polarization. It has been shown to attenuate the pro-inflammatory phenotype of pro-inflammatory (M1) macrophages and to facilitate the establishment of endotoxin tolerance. This immunomodulatory effect is partly attributed to the intracellular α-KG/succinate ratio, where a lower ratio inhibits prolyl hydroxylase (PHD)-dependent hydroxylation of IKK-β, suppressing NF-κB nuclear translocation (51). α-KG also promotes M2 macrophage activation through demethylation of H3K27 and activation of M2-related genes by enhancing the activity of Jmjd3, a histone demethylase (30) (**Figure 2**). Additionally, α-KG also serves as a cofactor for TET2, the main enzyme catalyzing the hydroxylation of 5-methylcytosine to 5-hydroxymethylcytosine (5hmC) in DNA. This TET2-mediated DNA hydroxymethylation activates transcription of anti-inflammatory genes such as Mrc1, Arg1, Chil3, and Retnla, thereby promoting the polarization of lung interstitial macrophages toward an anti-inflammatory phenotype (52) (**Figure 2**). In CD4 T cells, α-KG acts as a metabolic regulator that promotes Th1 cell differentiation by enhancing T-bet expression and activating mTORC1 signaling (53). Although α-KG enhances TET2 activity and promotes DNA demethylation at the Foxp3 locus, it paradoxically destabilizes Foxp3 expression. This leads to increased OXPHOS and lipid synthesis, thereby suppressing Treg differentiation (54). Conversely, in RA models, α-KG administered via polymeric nanoparticles has been reported to exert anti-inflammatory effects by suppressing pathogenic Th17 responses and promoting Treg differentiation, reducing joint inflammation (55). In monocytes from patients with SLE, elevated α-KG levels, driven by increased isocitrate dehydrogenase activity, act as cofactors for histone demethylases KDM6A/B, promoting H3K27 demethylation at interferon-stimulated gene (ISG) promoters and sustaining IFNα-induced gene expression (56).

## 4 Amino acid metabolism

### 4.1 Glutamine

Glutamine is an immunoregulatory nutrient and is the most abundant amino acid in the serum (57). SLC1A5, also known as alanine-serine-cysteine transporter 2 (ASCT2), is a transporter for neutral amino acids, including glutamine. *Slc1a5* ^-/-^CD4 T cells do not show impaired T-cell receptor-mediated activation, however, these T cells exhibit impaired Th1 and Th17 cell differentiation (58). Glutamine has the ability to regulate Th1 and Th2 balance. The addition of glutamine to the diet has been shown to favor Th1 cell responses over Th2 cells (59). Conversely, the addition of high concentrations of glutamine *in vitro* impairs Th2 cell differentiation, inhibiting inflammatory mediators such as leukotrienes, prostaglandins and platelet-activating factor, which are important for Th2 functions (60–62).

### 4.2 Glutamate

Glutamate is produced from glutamine by glutaminase (**Figure 1**). Glutaminase 1 expression is increased in Th17 cells, and the inhibition of glutaminase 1 reduces Th17 cell differentiation and ameliorates EAE and lupus-prone mice, MRL/*lpr* (63, 64). The inhibition of glutaminase 1 induces epigenetic changes in Th17 cells; however, these changes are not restored by a cell-permeable α-KG analog, dimethyl 2-ketoglutarate, suggesting a distinct mechanism of regulation of Th17 cells by glutaminase 1 (65). The inhibition of glutaminase 1 also reduced plasmablast differentiation in MRL/*lpr* mice (66). The glutaminase 1 inhibitor also reduced the number of activated B cells and Tfh cells in MRL/*lpr* mice (66, 67).

### 4.3 Aspartate

Aspartate is a non-essential amino acid that serves as a key intermediate linking cellular metabolism with immune cell function. It is primarily produced in the mitochondria by the transamination of oxaloacetate by glutamate (**Figure 1**), catalyzed by mitochondrial aspartate aminotransferase, and exported to the cytosol via the SLC25A12/13 transporters (68). Aspartate plays a central role in regulating TNF production in RA CD4 T cells (69). A deficiency in the production of mitochondrial aspartate disrupts NAD^+^ regeneration and ER stability, thereby promoting TNF translation (69). Supplementation with aspartate or NAD^+^ suppressed TNF release and tissue inflammation (69). In macrophages, aspartate acts as a metabolic regulator of inflammatory responses by promoting HIF-1α stabilization and promoting IL-1β production (70).

### 4.4 Tryptophan

Tryptophan is an essential amino acid and a precursor to kynurenine, serotonin, melatonin, and vitamin B3 (3). Kynurenine is a metabolite of tryptophan pathway used in the production of vitamin B3 (3). Metabolomic screening has identified an altered distribution of tryptophan metabolites in the feces of lupus-prone B6.Sle1.Sle2.Sle3 mice, including increased kynurenine levels, which are alleviated following antibiotic treatment. Interestingly, low dietary tryptophan intake ameliorates disease activity in these lupus-prone mice, whereas high dietary tryptophan intake exacerbates it.

## 5 One-carbon metabolism

### 5.1 Methionine

One-carbon metabolism serves as a crucial mechanism for cellular energy and production of vital signaling molecules, including single-carbon moieties (71). One-carbon metabolism includes folate and methionine metabolism. It also has important roles in DNA and RNA methylation, histone modification and redox homeostasis regulation (71). Intermediate metabolites in one-carbon metabolism, including methionine and SAM, mediate the T cell immune response.

Restriction of methionine inhibits Th17 cell differentiation and improves disease activity of EAE (72). Methionine is rapidly taken up by activated T cells and serves as the major substrate for the biosynthesis of SAM (72). Methylenetetrahydrofolate dehydrogenase 2 (MTHFD2), an enzyme of one-carbon metabolism, regulates *de novo* purine synthesis (73). In pathogenic Th17 cells, MTHFD2 prevents aberrant upregulation of Foxp3 and MTHFD2 deficiency promotes Treg cell differentiation (73).

### 5.2 SAM

SAM is the primary methyl donor for numerous cellular methylation reactions. Intracellular SAM levels are maintained within an optimal range by many homeostatic mechanisms (74). SAM is synthesized from methionine by methionine adenosyltransferase (MAT) (**Figure 1**), and MAT is inhibited by itaconate in Th17 cells (44). Reduced SAM levels decrease histone methylation at the *Il17a* promoter and reduce Th17 cell differentiation (72) (**Figure 2**). The SAM/SAH ratio is used as a methylation index, which means a key indicator of a cell’s methylation capacity (44). Thus some metabolites influence epigenetic changes.

## 6 Lipid metabolism

### 6.1 25-Hydroxycholesterol

25-OHC, produced by the enzyme cholesterol 25-hydroxylase (Ch25h) in activated CD4 T cells under IL-27 and TGF-β stimulation, acts as an immunomodulator. 25-OHC suppresses cholesterol biosynthesis in T cells by downregulating key enzymes such as HMG-CoA reductase via inhibition of the sterol regulatory element binding proteins (SREBP) 2 pathway, leading to selective apoptosis of proliferating T cells while sparing resting cells. Exogenous administration of 25-OHC by intraperitoneal injection reduces the severity of skin inflammation in a contact hypersensitivity model (75). Ch25h deficiency leads to excessive IL-1β production from macrophages, as 25-OHC normally suppresses IL-1β transcription and inflammasome activation (76). Ch25h-deficient mice show heightened sensitivity to septic shock and exacerbated EAE due to unrestrained AIM2-dependent inflammasome activity (77).

### 6.2 Sphingosine-1-phosphate

S1P biosynthesis involves sphingomyelinase-mediated ceramide generation from sphingomyelin, ceramidase conversion to sphingosine, and ATP-dependent phosphorylation by sphingosine kinases, localized to distinct subcellular compartments (78). This pathway bridges membrane lipid turnover to S1P’s immunomodulatory and pro-inflammatory signaling. S1P, via S1P receptor 1, is essential for T cell egress from the thymus and lymphoid organs, thereby controlling their circulation and tissue infiltration. S1P receptor modulators, such as fingolimod and siponimod, treat multiple sclerosis by binding S1PR1 on lymphocytes, prevent them from egress from lymph nodes and reducing their migration into the central nervous system (79). S1P levels were elevated in both SLE patients with active nephritis involvement and in the murine lupus models (80, 81). Sphingosine analogs ameliorate a murine lupus nephritis model, reducing T cell infiltration into the kidney (82). In RA-FLS, S1P stimulation promotes proliferation, migration, and production of IL-6 and MMP-3 (83).

## 7 Conclusion

The field of immunometabolism has illuminated the critical role of intracellular metabolites in modulating immune cell function and driving autoimmune pathogenesis. This review has highlighted representative immunometabolites from key metabolic pathways and their various effects on immune cells relevant to diseases such as RA and SLE. Targeting these immunometabolites could be an effective therapeutic strategy, as supported by findings from the animal models described above.

However, significant challenges remain. Given that certain metabolites such as lactate and succinate, have dual roles, careful consideration of the specific disease context and target cell type is essential. Technical hurdles include developing selective inhibitors of metabolic enzymes and designing delivery systems to achieve optimal metabolite concentrations within target immune cells. Furthermore, potential off-target effects and long-term consequences of metabolic interventions require thorough investigation.

Future research should focus on elucidating the complex interactions between immunometabolites and immune cell signaling. A more comprehensive understanding of the mechanisms underlying immunometabolite action could facilitate the development of highly specific and effective therapeutic strategies.
